# Thyrostimulin-TSHR signaling promotes the proliferation of NIH:OVCAR-3 ovarian cancer cells via trans-regulation of the EGFR pathway

**DOI:** 10.1038/srep27471

**Published:** 2016-06-07

**Authors:** Wei-Lin Huang, Zhongyou Li, Ting-Yu Lin, Sheng-Wen Wang, Fang-Ju Wu, Ching-Wei Luo

**Affiliations:** 1 Department of Life Sciences and Institute of Genome Sciences, National Yang-Ming University, Taipei 112, Taiwan

## Abstract

Gonadotropin signaling plays an indispensable role in ovarian cancer progression. We previously have demonstrated that thyrostimulin and thyroid-stimulating hormone receptor (TSHR), the most ancient glycoprotein hormone and receptor pair that evolved much earlier than the gonadotropin systems, co-exist in the ovary. However, whether thyrostimulin-driven TSHR activation contributes to ovarian cancer progression in a similar way to gonadotropin receptors has never been explored. In this study, we first found that TSHR is expressed in both rat normal ovarian surface epithelium and human epithelial ovarian cancers (EOCs). Using human NIH:OVCAR-3 as a cell model, we demonstrated that thyrostimulin promotes EOC cell proliferation as strongly as gonadotropins. Thyrostimulin treatment not only activated adenylyl cyclase and the subsequent PKA, MEK-ERK1/2 and PI3K-AKT signal cascades, but also trans-activated EGFR signaling. Signaling dissection using diverse inhibitors indicated that EOC cell proliferation driven by thyrostimulin-TSHR signaling is PKA independent, but does require the involvement of the MEK-ERK and PI3K-AKT signal cascades, which are activated mainly via the trans-activation of EGFR. Thus, not only have we proved that this ancient glycoprotein hormone system is involved in NIH:OVCAR-3 cell proliferation for the first time, but also that it may possibly become a novel oncotarget when studying ovarian cancer.

Glycoprotein hormones, which include follicle-stimulating hormone (FSH), luteinizing hormone (LH), chorionic gonadotropin (CG) and thyroid-stimulating hormone (TSH), have been found since 1940s and are known to be key endocrine hormones essential for many reproductive and metabolic functions in vertebrates^1^,^2^,^3^. These hormones are non-covalently linked heterodimers composed of two cystine knot glycoprotein subunits, a common α subunit and a distinct β subunit unique to each hormone. Of interest, in addition to these classic members, a sequencing search of the human genome has revealed two new glycoprotein hormone genes. These genes encode a novel glycoprotein α subunit, named GPA2, and a novel glycoprotein hormone β subunit, named GPB5. GPA2 and GPB5 can heterodimerize to form the fifth glycoprotein hormone that has been named thyrostimulin. This hormone has been demonstrated to be a more potent ligand than TSH when activating TSH receptor (TSHR)^4^. However, unlike the endocrine role of TSH, thyrostimulin exhibits a different and wider distribution across many tissues in vertebrates and has been suggested to act as a local but yet uncharacterized regulator^5^,^6^.

Although thyrostimulin is a newly discovered member, it plays an indispensable role in the evolution of glycoprotein hormones. Glycoprotein hormones co-evolve with their receptors and their presence can be traced back to invertebrates^7^,^8^,^9^. Intriguingly, genomic analyses have indicated that only one pair of *GPA2* and *GPB5* homologous genes and a *TSHR* homologous gene can be found in amphioxi and other invertebrates^9^,^10^; these findings indicate that the pair of thyrostimulin and TSHR evolved much earlier than other vertebrate-specific glycoprotein hormone systems and indeed is the most ancient glycoprotein hormone system. As a consequence of the absence of gonadal and thyroid glycoprotein hormones before the emergence of vertebrates, it can be speculated that the primitive roles of the thyrostimulin-TSHR pair may control both the metabolic rate and reproductive development in Metazoa in general.

Indeed, not only are thyrostimulin and TSHR capable of increasing serum thyroxine levels in mammals^4^, but many studies have also indicated that they are co-expressed in the mammalian ovary and may regulate some ovarian functions. For example, it has been shown that TSHR protein is expressed in the mature bovine corpus luteum, where it is postulated to be involved in the local synthesis of thyroid hormones or the local modulation of progesterone synthesis^11^. Aghajanova *et al*. also have revealed the existence of functional TSHR in mature human granulosa cells^12^. Following these findings, we have recently demonstrated that thyrostimulin, but not TSH, is expressed in the rat ovary. Thyrostimulin can be expressed in oocytes as a paracrine factor, allowing the TSHR-expressing granulosa cells to control their progesterone production^13^. Thus, similar to FSHR and LHR, the signaling associated with TSHR can also be involved in ovarian development.

Intriguingly, FSHR and LHR not only regulate the ovarian cycle and steroidogenesis in the ovary, but also are expressed in ovarian surface epithelium (OSE), where 90% of ovarian cancers originate^14^. Gonadotropin signals are able to promote proliferation, migration and anti-apoptosis in OSE in order to repair the ovarian surface rupture due to continuous ovulation^15^,^16^. However, they also can turn into key ovarian tumorigenic factors when there is excessive gonadotropin stimulation after menopause^17^. In the current study, we were surprised to find that, like the gonadotropin receptors, TSHR also exists in normal mammalian OSE and in human ovarian cancers. Taking into consideration the facts that gonadotropin signaling is the root to promote ovarian cancer progression and that the thyrostimulin-TSHR pair evolved much earlier than the gonadotropin signaling systems, we thus hypothesize that thyrostimulin-TSHR signaling is likely to be involved in ovarian cancer progression. In the present study we first explored the effects of thyrostimulin-TSHR signaling on ovarian cancer cell proliferation and then elucidated the signal flows among various downstreams involved.

## Results

### Expression of TSHR in ovarian surface epithelium and ovarian cancers

**Figure 1:**
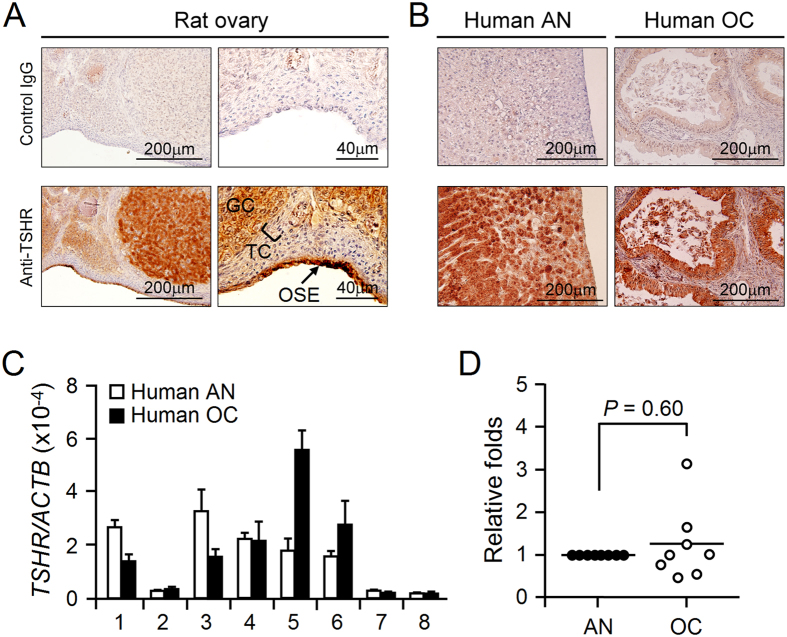
Expression of TSHR in rat normal OSE and human ovarian cancers. (**A**) Ovarian sections from mature rats and (**B**) representative sections from adjacent normal (AN) and cancer tissue (OC) samples that obtained from ovarian cancer patients were incubated with control rabbit IgG (upper panels) or anti-TSHR antibody (lower panels). The morphology of the cells was revealed by counter-staining with hematoxylin. GC: granulosa cell; TC: theca cell; OSE: ovarian surface epithelium. (**C**,**D**) The *TSHR* mRNA level in adjacent normal and cancer tissues of each patient was quantified. The β-actin level served as an internal control. The relative fold change in the *TSHR* transcript of the cancer tissue sample was normalized against its paired adjacent normal control for each patient.

The majority of ovarian cancers arise from OSE. To link TSHR signaling with ovarian cancer progression, we first tested whether TSHR is expressed in OSE. Using immunohistochemical staining, we found that a strong TSHR protein signal can be detected in normal rat OSE (Fig. 1A). Furthermore, we investigated eight cases of epithelial ovarian cancers (EOCs) using samples from the tumors and the corresponding normal tissues adjacent to the tumors; these were used to detect the protein level and transcript level of TSHR. We were able to detect the TSHR protein by staining in both the adjacent normal and tumor tissues (Fig. 1B). However, no significant difference was found when the *TSHR* transcript level was compared between the normal and cancerous samples (Fig. 1C,D). More supports were given by analyzing a gene expression array of ovarian serous adenocarcinoma from The Cancer Genome Atlas (TCGA; http://tcga-data.nci.nih.gov/tcga/) *in silico*^18^. Similar to our quantification results, although not significant, the transcript level of *TSHR* in the tumor cohort was relatively higher than the normal tissues (Supplementary Fig. S1A). Thus, TSHR is expressed in both normal OSE and in cancerous EOC; this is not unexpected because a similar expressional profile has also been observed previously for the gonadotropin receptors^19^,^20^. Of interest, we also found the appearance of *GPHA2* and *GPHB5*, the genes encoding the thyrostimulin heterodimer, in the array data, in which the transcript level of *GPHA2* was significantly elevated in the tumor cohort (Supplementary Fig. S1B,C)

**Figure 2:**
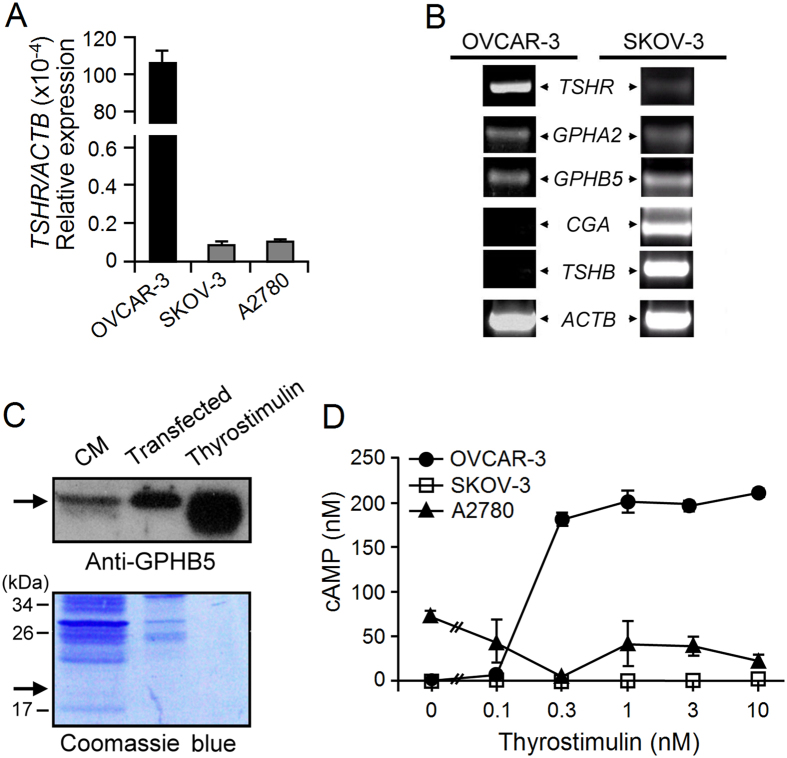
Expression of TSHR and thyrostimulin in ovarian cancer cell lines. (**A**) The *TSHR* transcript levels in different ovarian cancer cell lines were compared by real-time PCR quantification. The β-actin level served as a normalized control. (**B**) *TSHR* and the various glycoprotein hormone subunit genes, including *GPA2* and *GPB5* for thyrostimulin and *CGA* and *TSHB* for TSH, were PCR-amplified from the cDNA of ovarian cancer cells. The β-actin level served as a loading control. (**C**) Detection of endogenous thyrostimulin in NIH:OVCAR-3. Concentrated conditioned medium (CM) from NIH:OVCAR-3 cells was subjected to Western blotting using the anti-GPB5 antibody. Conditioned media from NIH:OVCAR-3 cells transfected with FLAG-GPA2/GPB5 and purified thyrostimulin (100 ng) were loaded and served as positive controls. The loading amounts are reflected in Coomassie blue staining. (**D**) Functional tests of endogenous TSHR in different cell lines. Cells were treated with graded doses of thyrostimulin for 16 h followed by the measurement of the amount of cAMP present in the conditioned media. Data are shown as the mean ± SD.

Next we screened several ovarian cancer cell lines in order to establish an appropriate cell model for thyrostimulin-TSHR signaling in ovarian cancers. As shown in Fig. 2A, NIH:OVCAR-3 expressed a much higher level of *TSHR* mRNA than SKOV-3 and A2780. We have reported previously that the ovary expresses thyrostimulin, but not TSH^13^. Interestingly, NIH:OVCAR-3 expressed only *GPHA2* and *GPHB5*, but no *CGA* and *TSHB*, the genes encoding TSH (Fig. 2B). In addition, the GPHB5 protein can be detected in the conditioned medium of NIH:OVCAR-3 (Fig. 2C), which suggests that these cells express thyrostimulin endogenously. Treatment with recombinant thyrostimulin increased the cAMP level in the cells dose-dependently, which is in contrast to the other two cell lines (Fig. 2D). These findings suggest that NIH:OVCAR-3 not only exhibits a similar transcript profile with respect to *TSHR* and thyrostimulin relative to that of the mammalian ovary, but also expresses functional TSHR protein. Thus, this cell line was chosen as an appropriate cell candidate for exploring the effects of thyrostimulin-TSHR signaling on ovarian cancers.

### Thyrostimulin-TSHR signaling promotes ovarian cancer cell proliferation

**Figure 3:**
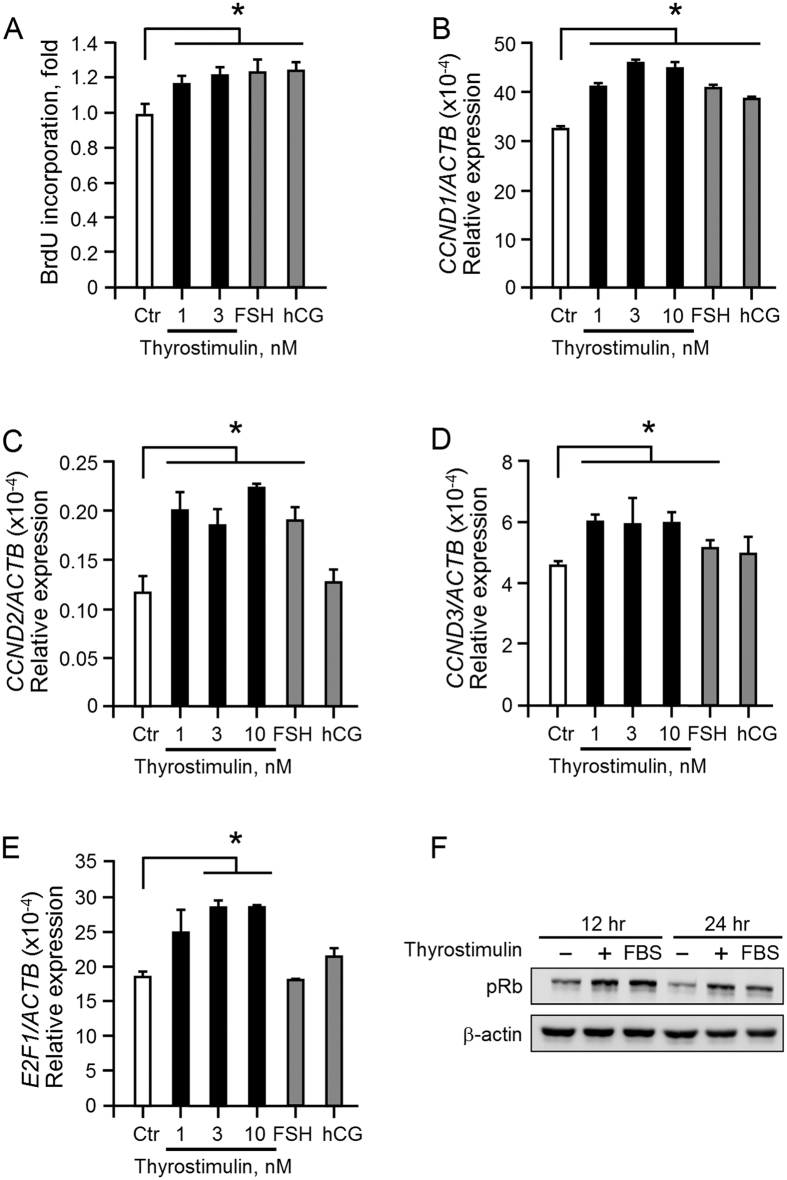
The effects of thyrostimulin on cell proliferation and proliferation-related markers. NIH: OVCAR-3 cells were treated with graded doses of thyrostimulin or with 3 nM of FSH or hCG for 24 h. (**A**) To measure BrdU incorporation, the cells were pulsed with BrdU for the last 4 h and the amount of incorporated BrdU in each group of cells was quantified. (**B**–**E**) To evaluate the expressional changes in various proliferation-related genes, treated cells were harvested for cDNA preparation. The transcript levels of the indicated proliferation-associated genes were quantified by real-time PCR and normalized against the level of β-actin in the same batch of cells. Data are shown as the mean ± SD. (**F**) Phosphorylated Rb without or with thyrostimulin treatment was detected by immunoblotting. The amount of β-actin protein present served as a loading control.

NIH:OVCAR-3 expresses both TSHR and gonadotropin receptors. We then compared the effect of thyrostimulin and various gonadotropins on ovarian cancer cell proliferation. Using the BrdU incorporation assay, thyrostimulin was found to promote NIH:OVCAR-3 cell proliferation in a manner as strong as gonadotropins (Fig. 3A). Furthermore, knockdown of *TSHR* decreased the proliferation rate of NIH:OVCAR-3 in a long-term culture (Supplementary Fig. S2). A subsequent evaluation of various cell cycle-associated genes confirmed that the transcript levels of *CCND1, CCND2, CCND3 and E2F1* were up-regulated after thyrostimulin treatment (Fig. 3B–E). Moreover, we also checked the phosphorylation levels of retinoblastoma protein (Rb), an indicator protein of proliferation. We found that phosphorylated Rb was also increased after thyrostimulin treatment (Fig. 3F). Therefore, these findings demonstrated that thyrostimulin is able to promote the proliferation of NIH: OVCAR-3 cells through their endogenously expressed TSHR. We also tried to perform the reverse set of experiments whereby TSHR was overexpressed in SKOV-3 and A2780. However, this was found to lead to the promotion of cell death during a long-term culture. Depending on the level and the type of cell tested, cAMP is known to exhibit opposing effects on cell growth^21^,^22^. Therefore, these findings may be explained as a side effect of the increased cAMP levels resulted from TSHR overexpression.

### Dissection of the signaling pathways activated by thyrostimulin in EOC cells

**Figure 4:**
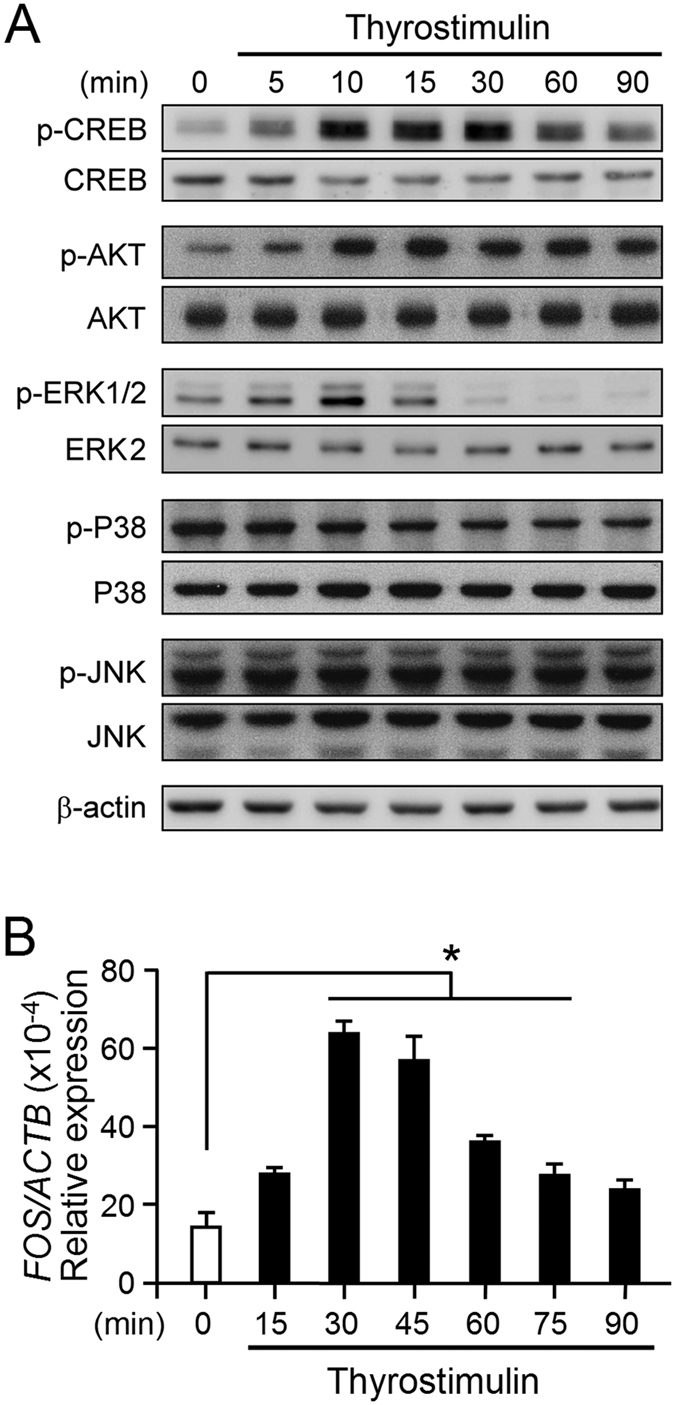
The signal cascades activated by thyrostimulin-TSHR signaling. NIH:OVCAR-3 cells were treated with thyrostimulin (3 nM) for the indicated time intervals. (**A**) The treated cells were lyzed and then immunoblotting was used to detect phosphorylated CREB, AKT, ERK1/2, P38 and JNK as well as their corresponding total protein levels. β-actin served as a loading control. (**B**) Treated cells at different time intervals were collected for cDNA preparation. The *c-FOS* transcript levels were then determined using real-time PCR. Data were normalized against β-actin and are shown as the mean ± SD.

TSHR is evolutionarily close to the gonadotropin receptors, which have been reported to be able to activate the PKA/CREB, MAPK and AKT pathways^20^,^23^,^24^,^25^,^26^. In order to characterize the potential TSHR downstream pathways in NIH:OVCAR-3, these cells were treated with thyrostimulin for the different time intervals and then the cell lysates were used to detect the phosphorylated levels of the indicated proteins by immunoblotting. We found that the phosphorylation levels of CREB, AKT and ERK, but not of p38 and JNK, were significantly elevated after treatment with thyrostimulin (Fig. 4A). The transcript of *FOS*, a downstream early nuclear response gene, was also elevated within 30 min after thyrostimulin treatment (Fig. 4B).

**Figure 5:**
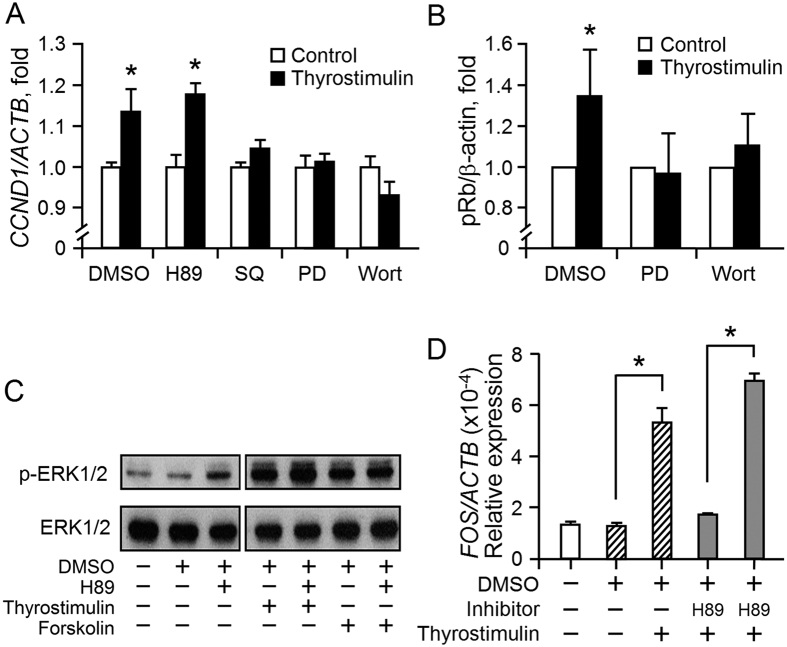
Dissection of the downstream effectors that mediate thyrostimulin-induced cell proliferation. (**A**) The effects of various signaling inhibitors on thyrostimulin-induced cell proliferation. Following 1h preincubation with the vehicle control, PKA inhibitor H89 (10 μM), adenylyl cyclase inhibitor SQ22536 (SQ, 50 μM), MEK inhibitor PD184352 (PD, 100 nM) or PI3K inhibitor wortmannin (Wort, 100 nM), NIH:OVCAR-3 cells were further treated with 10 nM thyrostimulin in the presence of above inhibitors for one day. The cells were harvested for cDNA preparation and the level of *CCND1* transcript was then quantified. Data were normalized against β-actin and the results are shown as the mean ± SD. (**B**) The cell lysates were subjected to Western blotting against phosphorylated Rb. Changes in the level of phosphorylated Rb were assessed by densitometer using the amount of β-actin protein present as a normalized control. Data are presented as the mean ± SE of three independent experiments. (**C**) Cells were pretreated with the vehicle control or PKA inhibitor H89, followed by treated with thyrostimulin or with forskolin as indicated for 10 min. The cell lysates were subjected to Western blotting to detect the amounts of phosphorylated ERK1/2 and total ERK1/2. (**D**) In addition, the cells, having undergone above treatments for 30 min, were used for quantification of *c-FOS* expression by real-time PCR. All data were normalized against the level of β-actin present and are shown as the mean ± SD.

Thyrostimulin promotes the proliferation of NIH:OVCAR-3 cells in a manner as strong as gonadotropins (Fig. 3). Thus we investigated the main signaling pathways that contribute to thyrostimulin-driven EOC cell proliferation. Using the *CCND1* level as a proliferation index, we found that thyrostimulin-induced cell proliferation can be abolished by co-incubation with the inhibitor against adenylyl cyclase (SQ22536), MEK (PD184352) or PI3K (wortmannin) (Fig. 5A). The effect of the MEK and PI3K inhibitors on thyrostimulin-driven cell proliferation was further confirmed by measuring the blockage level of Rb phosphorylation (Fig. 5B). These findings indicated that activation of the signaling cascades of adenylyl cyclase, MEK-ERK1/2 and PI3K-AKT is required for thyrostimulin-induced EOC cell proliferation. Intriguingly, although inhibition of adenylyl cyclase is able to dampen thyrostimulin-induced cell proliferation, we also noticed that blockage of PKA, the main downstream effector of cAMP, by adding inhibitor H89 has no equivalent effect (Fig. 5A). In addition, the PKA inhibitor showed no effect on ERK1/2 phosphorylation and *FOS* gene expression induced by thyrostimulin and on ERK1/2 phosphorylation induced by forskolin, an adenylyl cyclase activator (Fig. 5C,D). These findings suggest that, in NIH:OVCAR-3 cells, cAMP signaling promotes ERK phosphorylation and subsequent EOC cell proliferation via a PKA-independent route.

### Thyrostimulin-TSHR signaling promotes EOC cell proliferation through trans-regulation of EGFR

**Figure 6:**
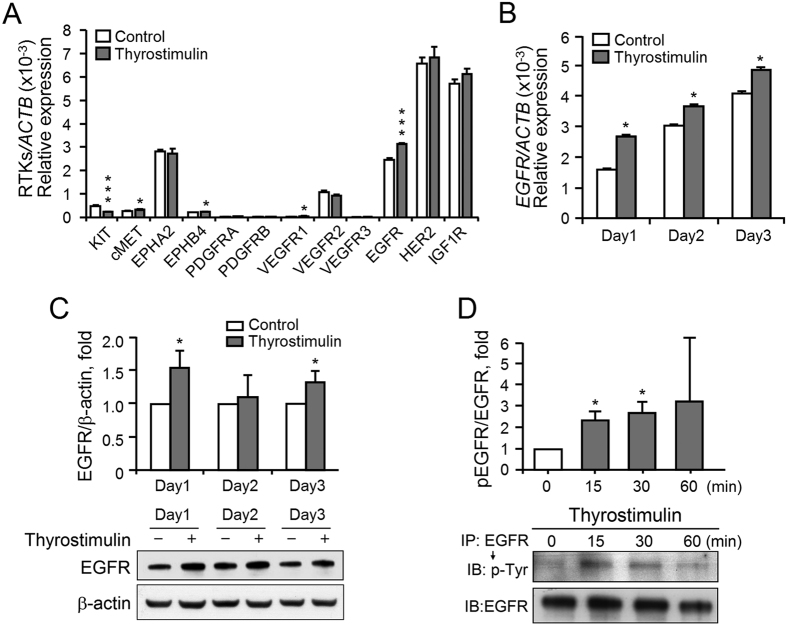
The effects of thyrostimulin-TSHR signaling on RTK expression and EGFR activation in NIH:OVCAR-3 cells. (**A**) The cells treated without or with thyrostimulin for 1 day were harvested for cDNA extraction and real-time quantification of the indicated RTKs. *P < 0.05; ***P < 0.001. To evaluate the trans-regulation of EGFR, the cells treated with or without thyrostimulin for the indicated time intervals were harvested for (**B**) the real-time quantification of the *EGFR* transcript, (**C**) the detection of the total amount of EGFR protein present or (**D**) the detection of the phosphorylated EGFR levels. For real-time PCR, data were normalized against β-actin and are shown as the mean ± SD. For Western blot quantification, the blots were assessed by densitometer using β-actin as a loading control. The results are shown as the mean ± SE of three independent experiments. One representative image of the blot was shown below.

The MEK-ERK1/2 and PI3K-AKT cascades are the common downstream signaling pathways of receptor tyrosine kinases (RTKs). Therefore, we hypothesized that thyrostimulin may activate these signal cascades via RTK regulation. To clarify this, the effect of thyrostimulin on transcript levels of twelve RTKs, which have been reported to be able to participate in ovarian tumor progression^27^,^28^,^29^,^30^,^31^,^32^, were investigated in NIH:OVCAR-3 cells. In the cells treated with thyrostimulin for 1 day, while the transcript level of *KIT* was decreased, the transcript levels of *MET, EPHB4, VEGFR1* and *EGFR* were elevated, with the change in *EGFR* being the most statistically significant (Fig. 6A). In addition, among the more abundant RTKs, including EPHA2, EGFR, HER2 and IGF1R, only the *EGFR* transcript was significantly elevated by thyrostimulin treatment. Therefore, we next explored the relationship between EGFR and thyrostimulin-TSHR signaling. Along with the culture days, we found that thyrostimulin-TSHR signaling promoted both the transcript level and the protein level of EGFR (Fig. 6B,C). Thyrostimulin-TSHR signaling not only regulated EGFR expression in long-term cultures, but also transiently activated EGFR signaling by increasing the phosphorylation level of EGFR (Fig. 6D). These findings show that activation of TSHR seems to contribute to an enhancement of EGFR signaling by increasing both the protein amount of EGFR present in the cells as well as the phosphorylation level of EGFR.

**Figure 7:**
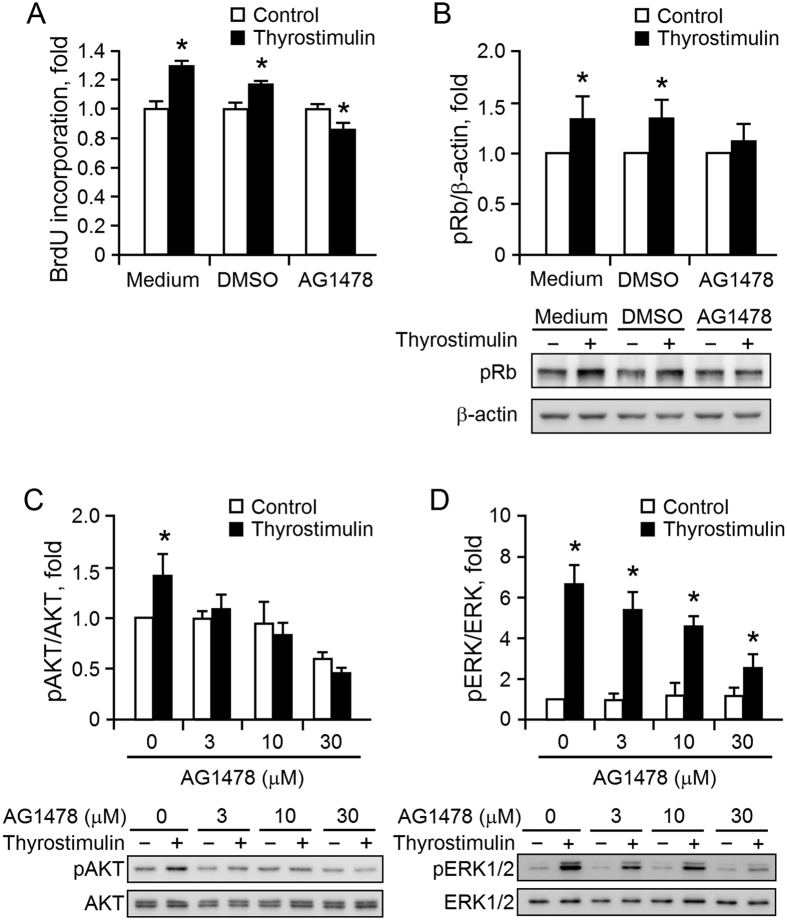
Thyrostimulin promotes EOC proliferation through the EGFR-mediated pathways. To carry out (**A**) BrdU cell proliferation assays and (**B**) the detection of phosphorylated Rb, NIH:OVCAR-3 cells preincubated with serum-free medium only, medium with the vehicle control or medium with EGFR inhibitor AG1478 (10 μM) were further treated without or with thyrostimulin for 1 day. To detect the effects of EGFR inhibitor on thyrostimulin-induced (**C**) AKT and (**D**) ERK activation, the cells preincubated with graded doses of EGFR inhibitor were then treated with or without thyrostimulin for 10 min. In order to evaluate any changes in the levels of above phosphorylated proteins, the blots were assessed by densitometer and normalized against the corresponding controls. Data are shown as the mean ± SE of three independent experiments. One representative image of the blot was shown below.

Activation of the EGFR pathway has been implicated in the growth and progression of ovarian cancers^33^. Surprisingly, when BrdU incorporation was investigated in parallel with changes in level of phosphorylated Rb, it was found that inhibition of EGFR activity resulted in a reversal of the promotion of thyrostimulin-driven NIH:OVCAR-3 cell proliferation (Fig. 7A,B). In addition, we also found that treatment with an EGFR inhibitor completely abolished thyrostimulin-induced AKT activation and also greatly inhibited thyrostimulin-induced ERK phosphorylation (Fig. 7C,D). These findings suggest that thyrostimulin-TSHR signaling is able to trans-activate the EGFR pathway and that this contributes significantly to thyrostimulin-mediated AKT and ERK activation, which subsequently controls EOC cell proliferation.

## Discussion

Hyperactivation of TSHR signaling has already been thought to be the root of toxic thyroid adenomas and differentiated thyroid carcinomas^34^,^35^. Although no direct link has been proposed between TSHR and ovarian cancers, an epidemiological study has shown that patients with hyperthyroidism have an 80% increased risk of ovarian cancer^36^; this implies that deregulation of TSHR signaling may contribute to ovarian tumorigenesis. By showing the promotion effect of thyrostimulin-TSHR signaling on EOC cell proliferation, to our knowledge this is the first report to demonstrate the direct involvement of TSHR signaling in certain aspect of ovarian tumor progression.

**Figure 8:**
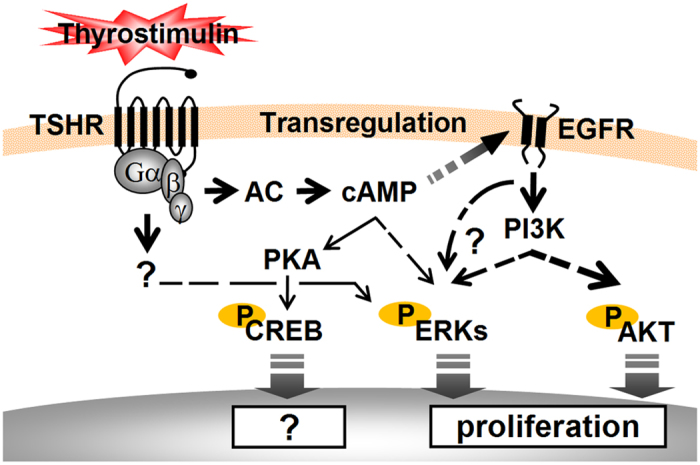
Thyrostimulin-TSHR signaling in OEC cells and the proposed regulatory mechanisms that mediate thyrostimulin-induced cell proliferation. Upon thyrostimulin binding, TSHR activates multiple downstream effectors to control ovarian cancer progression. Among these effectors, TSHR is able to activate the ERK and AKT signaling systems, mainly via the trans-activation of EGFR, to promote OEC cell proliferation.

Although the crosstalk between the TSHR downstream effectors awaits detailed studies, when this possibility is taken together with our current results, a relatively simplified model showing the main signal effectors of TSHR in the ovarian cancer cells and their underlying mechanisms that promote the proliferation of OEC cells can be developed and this is presented in Fig. 8. Upon thyrostimulin binding, TSHR interacts with cognate Gα proteins such as Gαs, which then activates adenylate cyclase to turn on the cAMP-dependent pathway. Promotion of EOC cell proliferation by thyrostimulin-TSHR signaling is cAMP dependent as an adenylyl cyclase inhibitor is able to block thyrostimulin-induced cell proliferation effectively (Fig. 5A). Via complicated crosstalks, this signal then induces activation of the CREB, ERKs and AKT pathways, which is reflected as increases in the phosphorylation levels of these proteins (Fig. 4A). However, further downstream analysis indicates that only the ERK pathway and AKT pathway, but not the PKA-CREB pathway, are involved in thyrostimulin-driven EOC cell proliferation (Fig. 5). It has been known that cAMP signaling can transactivate EGFR in many types of cancer cells^37^. Here we have also shown that thyrostimulin treatment is able to activate EGFR signaling (Fig. 6), potentially through the cAMP pathway. Interestingly, administration of an EGFR inhibitor effectively abolishes the phosphorylation of AKT and ERK as well as the promotion of cell proliferation brought about by thyrostimulin (Fig. 7). Although ERK is capable of being activated by PI3K in other cancers, such as breast cancer^38^, how it is activated by EGFR in our case needs more studies. Taken together, we can conclude that thyrostimulin-induced OEC cell proliferation is independent of the PKA pathway and would seem to mainly rely on crosstalk with EGFR, which subsequently activates the downstream AKT and ERK signaling systems.

In the present study, we have also noticed that the EGFR inhibitor is not able to totally inhibit thyrostimulin-induced ERK phosphorylation; this suggests that there might be other TSHR downstream effectors that contribute to the ERK activation. The MEK-ERK cascade mediates intracellular signals that are transduced by a wide variety of cell surface receptors. It is also a pathway well known to control cell proliferation in many cancers^39^. In addition to responding to EGFR activation, the MEK-ERK pathway has also been reported to be elevated directly by the hormone-induced cAMP signaling cascade^40^, which is the main downstream messenger of TSHR and gonadotropin receptors. Indeed, it has been reported that gonadotropins can activate ERK signaling via both the calcium-dependent and PKCδ-dependent mechanisms and this subsequently leads to the promotion of EOC cell proliferation and migration^25^. Like the activity of gonadotropin receptors in the ovarian cells, several studies have also reported that activation of TSHR can turn on calcium and PKC signaling in thyrocytes^41^,^42^. Therefore, although TSHR signaling has not yet been well characterized in the ovarian cancer cells, one may speculate that it can also activate the calcium and PKC pathways, which may then contribute to at least part of the thyrostimulin-induced ERK activation.

Thyrostimulin treatment also activates the PI3K-AKT cascade, which has been known to coordinate a complex signaling network involved in the promotion of cell proliferation in many types of cancers, including ovarian cancers^43^,^44^,^45^. For example, activated AKT can directly phosphorylate GSK3 to inhibit its activity^46^; this leads to the nuclear translocation of β-catenin to induce the expression of several cell cycle-related genes such as *CCND1* (symbol of Cyclin D1), which then promotes cell cycle progression via regulation of Rb phosphorylation. Thus, this may explain our observation that both the level of *CCND1* and the level of phospho-Rb are elevated in thyrostimulin-treated NIH:OVCAR-3 cells (Fig. 3). In addition, activation of the PI3K-AKT cascade also modulates numerous substrates that are related to cell growth, such as mTOR^47^, GLUT4 glucose transporter and various cyclin-dependent kinase inhibitors^48^,^49^,^50^. Therefore, it would be interesting to investigate further whether and how thyrostimulin-TSHR signaling regulates the activities of these proteins in ovarian cancers.

Of interest, although gonadotropins have also been reported to activate the MEK-ERK, PI3K-AKT and EGFR pathways, the interplay among these signaling molecules is quite distinct from our findings for thyrostimulin. Choi *et al*. demonstrated that gonadotropins activate the ERK and PI3K pathways and this then leads to an increase in EGFR expression in immortalized OSE cells. However, only a very mild or even no effect on EGFR up-regulation can be seen in ovarian cancer cell lines^26^. In contrast to their proposed model for gonadotropins, our findings show that the induction of AKT and ERK phosphorylation by thyrostimulin in NIH:OVCAR-3 cells is mainly through transactivation of EGFR (Fig. 7). This discrepancy may be due to differences in cell types used and/or the corresponding levels and types of the receptors in these cells. However, it is not possible to exclude the existence of synergistic regulation among the AKT, ERK and EGFR pathways.

In addition to EGFR, when we screened the RTKs commonly involved in ovarian tumor progression, we found that several RTKs with moderate expression levels can also be regulated by thyrostimulin-TSHR signaling in NIH:OVCAR 3 cells. For example, we observed that thyrostimulin treatment up-regulated the transcript levels of *MET* and *EPHB4* (Fig. 6A). Although the contributions of MET and EPHB4 to ovarian cancer remain relatively unclear as compared with the EGFR and VEGFR families, recent studies have observed that these two RTKs are regularly overexpressed in tumors from patients with ovarian cancers^51^,^52^. Hyperactivation of these two RTKs can promote ovarian tumor progression including survival, invasion and metastasis^53^,^54^. In contrast, thyrostimulin treatment reduced the expression of KIT (Fig. 6A). KIT is a well-documented oncogene in many cancers. Loss of KIT has been reported to be associated with progression in many cancers, such as breast cancer and thyroid epithelial cancer^55^,^56^. In these cases, KIT signaling may be involved in controlling the growth of normal epithelium and this function may be lost following malignant transformation. Although little is known about the function of KIT in ovarian cancer cells, the inhibition of KIT expression under thyrostimulin treatment might contribute to a reduction in KIT-mediated growth control and thus further promote ovarian cancer progression. Taken together, our preliminary findings provide hints that should help further studies into the involvement of thyrostimulin-TSHR signaling in various ovarian cancer progression traits, such as the capabilities of cancer cells to undergo anti-apoptosis, invasion and/or metastasis.

## Methods

### Ethical statements and clinical materials

The 8 paired EOCs and matched adjacent normal tissues were collected from patients who underwent surgery at Chang Gung Memorial Hospital Linkou Medical Center. All the clinical materials were frozen and stored at −80 °C for further real-time quantitative PCR and immunohistochemical analysis. Written informed consent was signed and obtained from all subjects. All experimental protocols were approved by the research ethics committee of Chang Gung Memorial Hospital Linkou Medical Center, Chang Gung University (approval number 97–0675 C). The methods were carried out in accordance with the approved guidelines. The microarray data of human ovarian serous cystadenocarcinoma from TCGA were processed and analyzed by the Cancer Genomics Browser (ID: TCGA_ TCGA_OV_G4502A_07_2) (https://genome-cancer.ucsc.edu)^57^.

### Hormones, reagents and plasmids

The full-length *GPHA2* and *GPHB5* cDNAs were amplified from human ovarian cDNA (CLONTECH, Palo Alto, CA) and subcloned into the bipromoter expression vector pBudCE4.1 (Invitrogen). GPHA2 was tagged with an N-terminal FLAG to facilitate protein purification. Recombinant human thyrostimulin was purified from transfected 293T conditioned media as described previously^13^. Human FSH and hCG were purchased from Calbiochem (La Jolla, CA). DMEM medium, RPMI 1640 medium, penicillin, streptomycin, glutamine and zeocin were purchased from Invitrogen (Carlsbad, CA). Rabbit anti-TSHR antibody was purchased from Novus Biologicals (NBP1-87823, Littleton, CO). Rabbit anti-CREB antibody was purchased from (Millipore, Billerica, MA). Rabbit anti-ERK2 antibody was from Santa Cruz Biotechnology (St. Cruz, CA). Antibodies against phospho-AKT, phospho-CREB, phospho-Rb, phospho-ERK1/2, phospho-P38, phospho-JNK, phospho-tyrosine and total form of AKT, P38, JNK or EGFR were purchased from Cell Signaling Technology (Danvers, MA). Other chemicals unless noted otherwise were purchased from Sigma (St. Louis, MO).

For knockdown of TSHR, pLKO.1-shGFP and pLKO.1-shTSHR were purchased from the National Core Facility for Manipulation of Gene Function by RNAi, miRNA, miRNA sponges, and CRISPR/Genomic Research Center, Academia Sinica. The recombinant lentiviruses were obtained by cotransfection with pCMVΔR8.91, pMD.G and pLKO.1-shGFP or pLKO.1-shTSHR HEK-293T through Turbofect transfection reagent. The condition media were collected and used for further NIH:OVCAR-3 cell infection.

### Immunohistochemical staining, Western blotting and immunoprecipitation

For immunohistochemical staining, the ovaries from mature rats and the clinical materials of ovarian cancers and the paired adjacent normal tissues were fixed by Bouin’s solution following paraffin embedding. The antigens on 5 μm-thick sections were retrieved by Target Retrieval Solution, pH 9 (DAKO). For the performance of immunohistochmeistry, rabbit anti-TSHR antibody or normal IgG (Cell Signaling Technology) at the same concentration was used. The staining was performed using the Universal LSAB^TM+^ Kit/HRP (DAKO) and NovaRed HRP substrate kit (VECTOR) according to the manufacturers’ instructions.

To detect the endogenously expressed thyrostimulin in NIH:OVCAR-3 cells, the cells were cultured in the serum-free media for 3 days and the conditioned media were then collected and concentrated. To assess the changes in phosphorylation levels of downstream effectors, NIH:OVCAR-3 cells were preincubated in serum-free conditions for 16 hr before being treated with effector inhibitors and/or thyrostimulin for different time intervals. After treatment, the cell lysates were subjected to Western blotting using antibodies specific for phosphorylated targets, their total form and/or β-actin.

To evaluate the thyrostimulin-induced EGFR phosphorylation, NIH:OVCAR-3 cells were preincubated in serum-free conditions for 16 hr before being treated with thyrostimulin for different time intervals. The cells were washed and lyzed with ice-cold NP-40-containing lysis buffer followed by sonication for 5 sec and centrifugation. Magnetic protein G beads (GE Healthcare) preincubated with anti-EGFR antibody were incubated with the supernatants overnight at 4 °C to precipitate EGFR. The beads were washed ice-cold NP-40-containing lysis buffer and boiled with the protein sample buffer. The elute was subjected to Western blotting and probed with anti-phospho-tyrosine antibody to reveal the phosphorylated EGFR levels.

### cAMP assay

To demonstrate the existence of functional TSHR in ovarian caner cell lines, NIH:OVCAR-3, SKOV-3 and A2780 cells cultured in serum-free media supplemented 0.25 mM 3-isobutyl-1-methylxanthine were treated with graded doses of thyrostimulin for 16 h. The amounts of cAMP in the media were determined using the cAMP-Glo Assay kit following the manufacturer’s instructions (Promega, Madison, WI).

### Cell proliferation assays

NIH:OVCAR-3 cells were trypsinized, counted, resuspended with FBS-containing media. For BrdU incorporation analysis, 1.25 × 10^5^ NIH: OVCAR-3 cells were plated into each well of 48-well plates and incubated for one day for attachment. Following attachment, the cells were washed, starved with serum-free medium and then treated without or with thyrostimulin and/or effector inhibitors for 24 h. 10 μM BrdU was added into medium for the last 4 h. To measure the amount of incorporated BrdU, BrdU cell proliferation assay kit (Cell Signaling Technology) was used following the manufacturer’s instructions. For long-term culture to observe the knockdown effect of *TSHR* on cell proliferation, the cells were resuspended with FBS-containing media and then seeded in 48-well plates (1500 cells/well). At indicated intervals, 10% medium volume of AlamarBlue (AbD Serotec) was directly added in the wells and the plates were incubated for 3 h at 37 °C. The fluorescent signal is monitored using an excitation wavelength at 560 nm and emission wavelength at 590 nm according to the manufacture’s protocols. All experiments were performed in a triplicate manner.

### cDNA preparation and gene quantification

For cDNA preparation, the total RNA from cancer cell lines or clinical materials were collected and extracted by TRIzol (Invitrogen) according to the manufacturer instructions. The cDNA of total mRNA were synthesized by High-Capacity cDNA Reverse Transcription Kits (Life technology, Grand island, NY) with oligo-dT primer. The primer pairs for the semi-quantitative PCR were listed as follows: human *TSHR* forward, GTGAATGCTTTTCAGGGACTATG; human *TSHR* reverse, GTCCAGGTGTTTCTTGCTATCAG. Human *GPHA2* forward, AGGCAGTCATCCCAGGC; human *GPHA2* reverse, TCACTTCGCACTGTCACATTGA. Human *GPHB5* forward, TGACTGTCAAGCTGCCCAACT; human *GPHB5* reverse, GGATGGCCACGGGATAGGTGTAGA. Human *CGA* forward, GCTCCTGATGTGCAGGAT; human *CGA* reverse, TTAAGATTTGTGATAATA. Human *TSHB* forward, TTTGTATTCCAACTGAG; human *TSHB* reverse, TTAGACAGAAAATCCTAC. Human *ACTB* forward, TGACAGACTACCTCATGAAGATCC; human *ACTB* reverse, CTGCT TGCTGATCCACATCTG. For the quantitative real-time PCR, Power SYBR Green PCR Master Mix was used and the primer pairs were listed in Supplementary Table S1.

### Data analysis

For real-time quantitative PCR, cell proliferation and cAMP assays, data are presented as the mean ± SD of triplicate cultures or samples. At least three individual repeated experiments were carried out and these showed similar results. For the quantification data that were shown in Western blotting, the blots from at least three independent experiments with the same treatment conditions were assessed by densitometer and are presented as the mean ± SE. For Western blots that were not subjected to quantification, at least three similar experiments were carried out and these showed the same tendency. Student’s t-test (unpaired, two-tailed) was used to compare differences between two groups and One-way ANOVA was used to compare differences between multiple groups. The Mann-Whitney test was applied to analyze the differences in the transformed gene values in ovarian cancer microarray. Significance was accepted at *P* < 0.05 and is indicated by asterisks unless otherwise noted.

## Additional Information

**How to cite this article**: Huang, W.-L. *et al*. Thyrostimulin-TSHR signaling promotes the proliferation of NIH:OVCAR-3 ovarian cancer cells via trans-regulation of the EGFR pathway. *Sci. Rep.* **6**, 27471; doi: 10.1038/srep27471 (2016).

## Supplementary Material

Supplementary Information
