# Supplementary material for: Thyrostimulin-TSHR signaling promotes the proliferation of NIH:OVCAR-3 ovarian cancer cells via trans-regulation of the EGFR pathway
Source: Sci Rep. 2016 Jun 7;6:27471. doi: 10.1038/srep27471 (PMC4895341; doi:10.1038/srep27471)
Supplement: Supplementary Information [file srep27471-s1.pdf]

Thyrostimulin-TSHR signaling promotes the proliferation of NIH:OVCAR-3 ovarian cancer cells via trans-regulation of the EGFR pathway

Wei-Lin Huang[1], Zhongyou Li[1], Ting-Yu Lin, Sheng-Wen Wang, Fang-Ju Wu and Ching-Wei Luo*

Department of Life Sciences and Institute of Genome Sciences, National Yang-Ming University, Taipei 112, Taiwan.

[1] WL Huang and Z Li contributed equally to this work.

*** Correspondence:**

Ching-Wei Luo, Department of Life Sciences and Institute of Genome Sciences, National Yang-Ming University, 155 Li-Nong Street, Section 2, Beitou, Taipei 112, Taiwan. Tel: 886-2-28267185; Fax: 886-2-28202449. Email: cwluo@ym.edu.tw

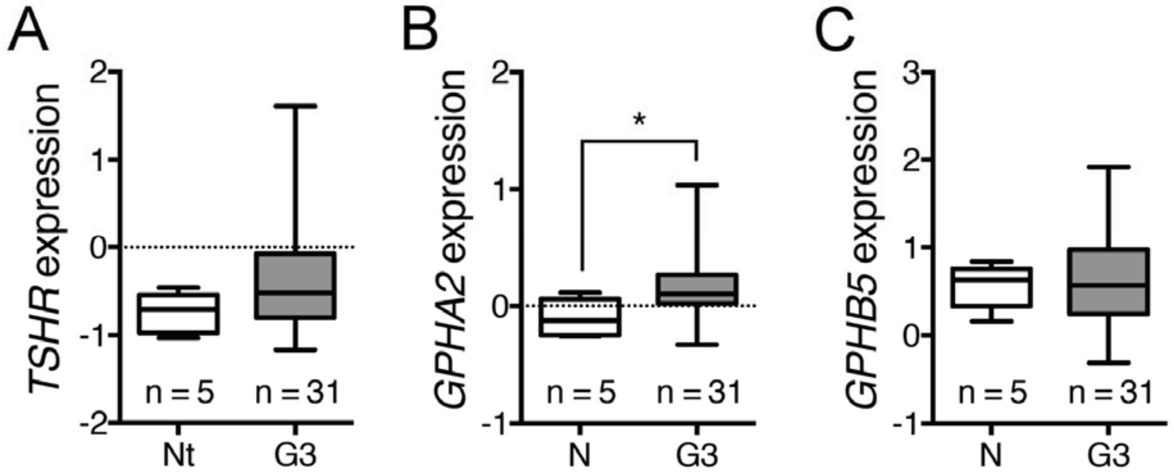

Supplementary Fig. S1. The expression profiles of *TSHR*, *GPHA2* and *GPHB5* in the human ovarian cancer samples.

The transcript values of (A) *TSHR*, (B) *GPHA2* and (C) *GPHB5* were extracted from a microarray dataset of human ovarian cancer samples (ID: TCGA_ TCGA_OV_G4502A_07_2) and compared between normal tissues (Nt) and grade III ovarian cancer (G3). The gene values were normalized and log_2 transformed by UCSC Cancer Genomics Browser. The boxes represent the interquartile range; the horizontal lines in the boxes represent the median; the whiskers represent the minimum and maximum.

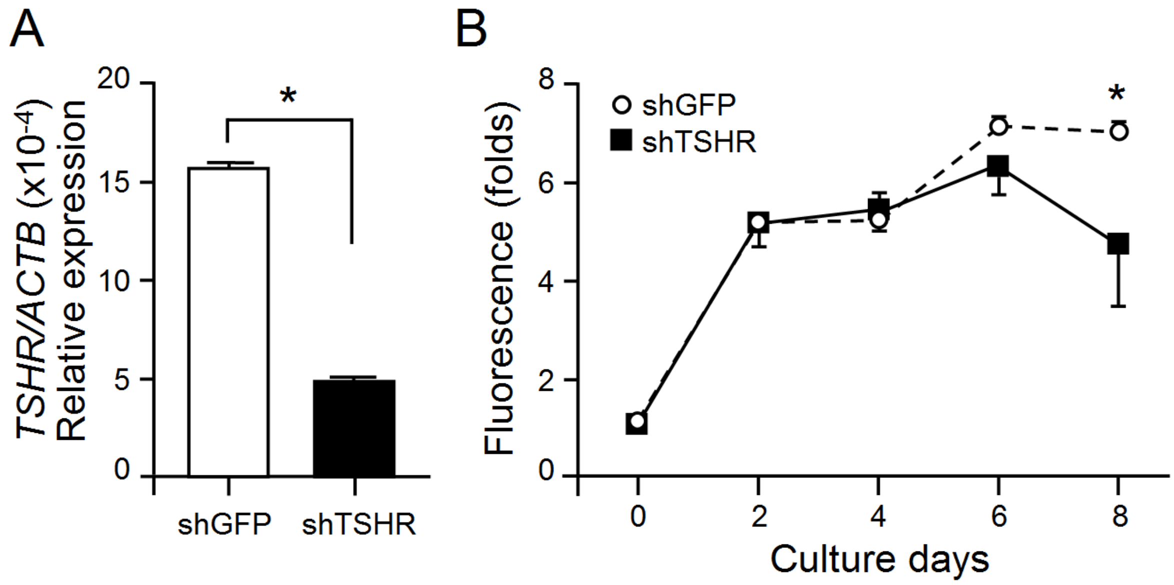

Supplementary Fig. S2. Knockdown of *THSR* dampens the proliferation of NIH:OVCAR-3 cells.

(A) NIH:OVCAR-3 cells were infected with a lentiviral shRNA targeting *TSHR* or with a *eGFP* lentiviral control for 24 h. The cells were selected with puromycin and then recultured in fresh media. The *TSHR* levels were determined by real-time PCR quantification. β-actin served as a normalized control. (B) Cell proliferation rates of the *eGFP*-knockdown cells and the *TSHR*-knockdown cells were compared by AlamarBlue assay. The fluorescence value of the cells on day 0 served as the one-fold control. Data are shown as the mean ± SD.

Supplementary Table S1. The primer pairs for real-time quantitative PCR.

Gene symbol	**Direction**	**Sequence**
TSHR	Fw	ACCCAGGGGACAAAGATACC
	Rv	GAATGGATTGGCACAGGAGT
EGFR	Fw	ACCCCCTCCTTACGCTTTGT
	Rv	TGGCCAGAGCTGTAAGTGCTT
IGF1R	Fw	AGTGAGGTTGAGGTGAGAGGTTTG
	Rv	CCGCCTTCTGGTTTGATTTTT
ERBB2	Fw	CCCCAAAGCCAACAAAGAAA
	Rv	CCGTGGATGTCAGGCAGAT
PDGFRA	Fw	TGTTGGTGCTTTGCATTTTGAT
	Rv	TCAGGTGGGAGCATTTGTTAGG
PDGFRB	Fw	GGGACAAAGAGGGCAAATGA
	Rv	ATAACTGTCCTCACTGTCCATTCTGT
VEGFR1	Fw	GCTGGCTCTGTTTGATGCTATTT
	Rv	GTTGCCTCTCCAGCTTCTGACT
VEGFR2	Fw	TTTTTGCCCTTGTTCTGTCCTT
	Rv	TCATTGTTCCCAGCATTTCACA
VEGFR3	Fw	CATGACCCCAACGACCTACAA
	Rv	ATGCCTGCTCTCTATCTGCTCAA
MET	Fw	GAATAGCCACCCTGAGCAGAAC
	Rv	ACACCACATGCACTATACAGTAGCAA
KIT	Fw	CCCTTCCTCACTGCCCAATA
	Rv	GGAAACCATAAAGGCAACATACCT
EPHA2	Fw	TGGCAACTTGGCGGTGAT
	Rv	CGGGCACGCTGGTTCTT
EPHB4	Fw	CCTGTTTCACTATGGCCTCCTTT
	Rv	GGGATGACCAAGGCACTGTT
ACTB	Fw	TCCTCCTGAGCGCAAG
	Rv	CTGCTTGCTGATCCACATCT
